# Plasma dynamic monitoring of soluble c-Met level for EGFR-TKI treatment in advanced non-small cell lung cancer

**DOI:** 10.18632/oncotarget.9425

**Published:** 2016-05-18

**Authors:** Hong-Fei Gao, Jin-Ji Yang, Zhi-Hong Chen, Xu-Chao Zhang, Hong-Hong Yan, Wei-Bang Guo, Qing Zhou, Lou-Ying Gou, Zhong-Yi Dong, Yi-Long Wu

**Affiliations:** ^1^ Guangdong Cardiovascular Institute, Guangdong General Hospital & Guangdong Academy of Medical Sciences, Guangzhou, China; ^2^ Guangdong Lung Cancer Institute, Guangdong General Hospital & Guangdong Academy of Medical Sciences, Guangzhou, China

**Keywords:** soluble c-Met, EGFR-TKI, NSCLC

## Abstract

**Background:**

The activation of c-Met has been associated with both primary and acquired resistance to EGFR-TKI therapy in NSCLC patients. Thus, c-Met status during EGFR-TKI therapy should receive much attention.

**Results:**

Forty-nine patients were selected as training cohort and 52 cases as validation cohort. With disease progression, IHC results showed that 37 (75.5%) of the patients were tissue c-Met-negative, and 12 (24.5%) were tissue c-Met-positive. There was a statistically significant difference in the dynamic change in soluble c-Met between the tissue c-Met-negative and c-Met-positive groups (*P* = 0.002). Patients with a baseline soluble c-Met level >766 ng/ml showed inferior median progression-free survival (PFS; 10.2 *vs*. 14.0 months; *P* = 0.003) after EGFR-TKI treatment. Multivariate Cox proportional hazards model analyses demonstrated that the soluble c-Met level was an independent prognostic factor for PFS after EGFR-TKI treatment (*P* = 0.009; hazard ratio: 3.583; 95% confidence interval: 1.379-9.312). In the validation cohort, patients with soluble c-Met levels >766 ng/ml were also determined to have significant short median PFS after EGFR-TKI treatment (6.8 *vs*. 14.5 months, *P* < 0.001).

**Patients and Methods:**

We retrospectively investigated the dynamic change in the soluble c-Met level in plasma and its relationship with clinical outcomes of EGFR-TKI therapy in advanced NSCLC. Immunohistochemistry (IHC) was used to assess the expression of c-Met in the resistant tissue. Plasma c-Met levels were assayed in duplicate using a human soluble c-Met quantitative enzyme-linked immunosorbent assay (ELISA) kit.

**Conclusions:**

Quantitatively determining the soluble c-Met level in plasma by ELISA might provide a non-invasive and sensitive method to predict EGFR-TKI prognosis.

## INTRODUCTION

Activating mutations in epidermal growth factor receptor (EGFR), particularly the exon 19 del and exon 21 L858R mutations, are associated with sensitivity to EGFR tyrosine kinase inhibitor (EGFR-TKI) therapy [1–3]. However, despite the dramatic benefits from EGFR-TKI in this genetically defined cohort, almost all patients will ultimately develop resistance to EGFR-TKI, in whom dysregulation of c-Met is observed in up to 20% of resistance cases [4, 5]. The c-Met abnormities include c-Met protein overexpression, gene amplification or mutation [6–8]. Several clinical trials have demonstrated that c-Met protein overexpression could be used as a biomarker for acquired resistance to EGFR-TKI, and a combination of c-Met and EGFR dual inhibitory strategies showed a synergistic benefit in c-Met protein overexpression patients with acquired resistance to EGFR-TKI [9–11]. Furthermore, de novo c-Met dysregulation subclones might be present in some lung cancers but not at a frequency that is high enough for detection until the proper EGFR-TKI therapy-associated selective pressures are applied [12, 13]. All of these studies suggest that c-Met status during EGFR-TKI therapy should receive much attention.

Clinically, c-Met status is most often determined in tissue by immunohistochemistry (IHC), which evaluates protein expression, or by fluorescence *in situ* hybridization (FISH) assays that determine gene amplification [14]. However, both of these assays are cell-based and require tissue sample preparation. For the detection of c-Met expression, tumor tissue is the most usual sample source. However, for most advanced NSCLC cases, detection is always limited by insufficient tissue or the dynamic monitoring of c-Met status. Thus, exploring supplementary samples and noninvasive assays for c-Met detection is needed.

The c-Met is a transmembrane protein consisting of an α- and a β-subunit linked together by a disulfide bond. Extracellular fragments of c-Met protein may be shed from the cell surface through a proteolytic process, facilitating the generation of soluble truncated c-Met protein, which can be easily measured in human blood [15–19]. A significant and direct correlation between the shedding of soluble c-Met and the total amount of tissue c-Met has been established [18]. Blood is a representative fresh and real-time sample that, as a noninvasive method, could also facilitate the dynamic monitoring of c-Met during therapy. In our previous research, we compared tissue c-Met protein expression by IHC with soluble c-Met levels *via* an enzyme-linked immunosorbent assay (ELISA) in 198 advanced NSCLC patients. We found a statistically significant correlation: patients whose tumor tissue showed c-Met positivity also tended to have elevated soluble c-Met levels in plasma. A plasma c-Met level of 766 ng/ml showed moderate specificity and sensitivity in predicting tissue c-Met protein expression. A high level of soluble c-Met was associated with a poor prognosis (detailed data not shown).

The role of soluble c-Met during EGFR-TKI therapy is unclear. Therefore, the purpose of this study was to examine the dynamic change in the plasma soluble c-Met level in advanced NSCLC patients receiving EGFR-TKI treatment using a human soluble c-Met quantitative ELISA kit. We evaluated the potential usefulness of determining soluble c-Met levels to predict the prognosis of EGFR-TKI treatment.

## RESULTS

### Patient characteristics

Forty-nine patients were selected as training cohort and 52 cases as validation cohort for prognosis analysis. In the training cohort, most of these patients had adenocarcinoma histology (47/49; 95.9%), with progression-free survival (PFS) after EGFR-TKI > 6 months (46/49; 93.9%). In the validation cohort, 98.1% (51/52) of these patients had adenocarcinoma histology. The clinicopathological features are summarized in Table 1.

**Table 1:** Patient characteristics

	Study Patients *n*(%)	Validation Patients *n*(%)
**N**	49	52
**Median age, y**	56 (31-80)	60 (37-79)
**Sex**		
Male	18 (36.7)	24 (46.2)
Female	31 (63.3)	28 (53.8)
**Smoking**		
Smoker	7 (14.3)	12(23.1)
Never smoker	42 (85.7)	40(76.9)
**Number of metastatic sites**		
1	20 (40.8)	28(53.8)
≥2	29 (59.2)	24(46.2)
**EGFR mutation**		
19del	34 (69.4)	31(59.6)
21L858R	15 (30.6)	21(40.4)
**EGFR-TKI**		
First-line	37 (75.5)	43(82.7)
Second-line or beyond	12 (24.5)	9(17.3)

### Association between the plasma soluble c-Met level and tissue c-Met status

**Figure 1:**
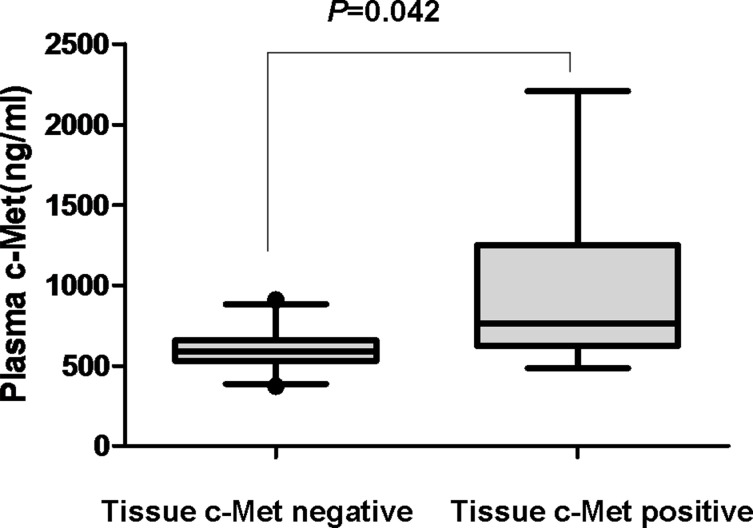
Plasma c-Met concentration according to tissue c-Met status with PD in the training cohort

With disease progression (PD), all patients in the training cohort underwent rebiopsy after resistance to EGFR-TKI therapy. Tissue c-Met protein expression was evaluated by IHC according to H score criteria. Of the 49 patients, 37 (75.5%) were tissue c-Met-negative, and 12 (24.5%) were tissue c-Met-positive. We observed a positive correlation between the soluble c-Met level with PD and tissue c-Met status in resistant tumors. We observed no other correlations between plasma c-Met levels and the clinicopathological parameters, including age, sex, histological type, EGFR mutation type, and smoking history. The average plasma c-Met concentration was 601.55 ± 120.94 ng/ml in the tissue c-Met-negative group, which was significantly lower than 986.42 ± 578.36 ng/ml in the tissue c-Met-positive group (*P* = 0.042; Figure 1). Our laboratory previously showed that a soluble c-Met level > 766 ng/ml could predict tissue c-Met positivity and was associated with poor survival in advanced NSCLC. Thus, we used this cut-off value in the present study. The concordance between IHC and ELISA was 79.6%. Soluble c-Met levels above 766 ng/ml at the time of PD were associated with a positive c-Met status of the resistant tumor in 6 of 12 patients (50.0%). Plasma c-Met levels below 766 ng/ml at the time of PD were associated with a negative c-Met status of the resistant tumor in 33 of 37 patients (89.2%) (*P* = 0.008; Table 2).

**Table 2:** Comparison of c-Met status by ELISA and IHC in the training cohort

	Tissue c-Met-positive	Tissue c-Met-negative
Plasma c-Met >766 ng/ml	6	4
Plasma c-Met <766 ng/ml	6	33

### Dynamic change in the soluble c-Met level in plasma during EGFR-TKI treatment

**Figure 2:**
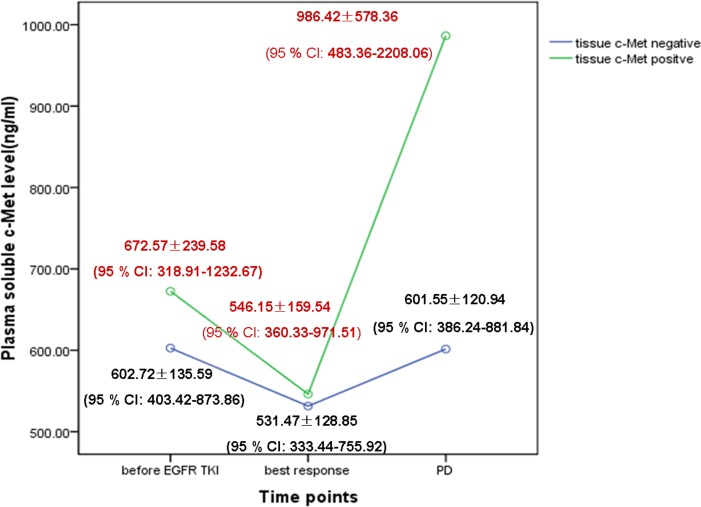
Dynamic change in the soluble c-Met level in plasma during EGFR-TKI treatment according to tissue c-Met protein expression with PD in the training cohort

Soluble c-Met changes during EGFR-TKI treatment were measured and analyzed using repeated measures analysis of variance (ANOVA). The average plasma c-Met was 619.83 ± 166.92 ng/ml (95% confidence interval [CI]: 571.88 - 667.77) before EGFR-TKI treatment, decreased to 535.07 ± 135.37 ng/ml (95% CI: 496.19 - 573.95) at the time of the best tumor response, and then increased to 695.80 ± 339.98 ng/ml (95% CI: 598.15 - 793.46) with PD (*P* = 0.001). To identify the effect of soluble c-Met dynamic change on the resistance to EGFR-TKI, we divided the 49 patients into two groups according to c-Met status in the tumor tissue with PD. In tissue c-Met-positive patients, we found significantly elevated soluble c-Met levels, and there was a statistically significant difference in the dynamic change in the soluble c-Met level between the tissue c-Met-positive and c-Met-negative groups (*P* = 0.002; detailed data shown in Figure 2).

Among tissue c-met-positive patients, 4 (33.3%, 4/12) had elevated pre-EGFR-TKI soluble c-Met levels (> 766 ng/ml), and 6 (50%, 6/12) had elevated soluble c-Met levels with PD. In the tissue c-Met-negative cohort, only 3 patients (8.1%, 3/37) had elevated soluble c-Met levels pre-EGFR-TKI treatment, and 4 patients (10.8%, 4/37) had elevated soluble c-Met with PD.

Soluble c-Met levels > 766 ng/ml before EGFR-TKI treatment were associated with a positive c-Met status of the resistant tumor in 4 of 12 patients (33.3%). Plasma c-Met levels < 766 ng/ml before EGFR-TKI treatment were associated with a negative c-Met status of the resistant tumor in 34 of 37 patients (91.9%, *P*= 0.051).

### A high soluble c-Met level in pre-EGFR-TKI plasma predicts short PFS after EGFR-TKI treatment

**Figure 3:**
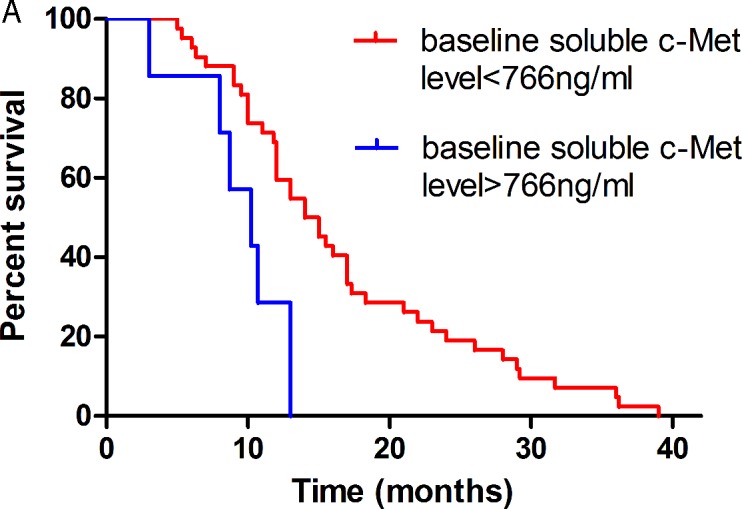

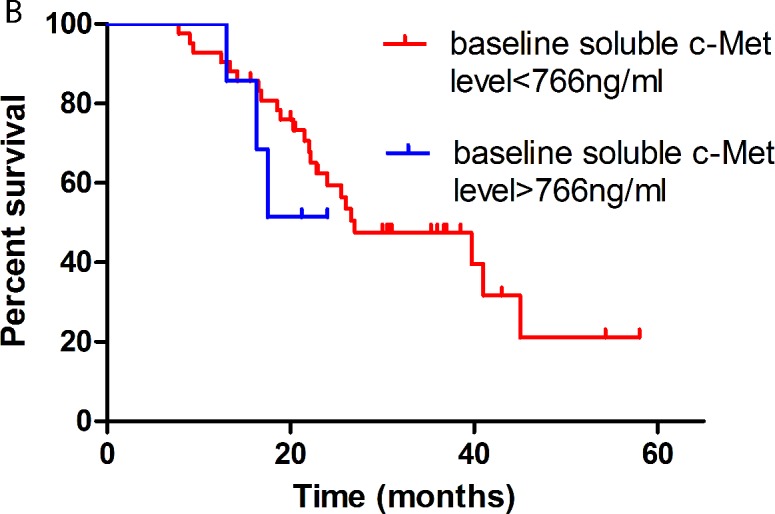

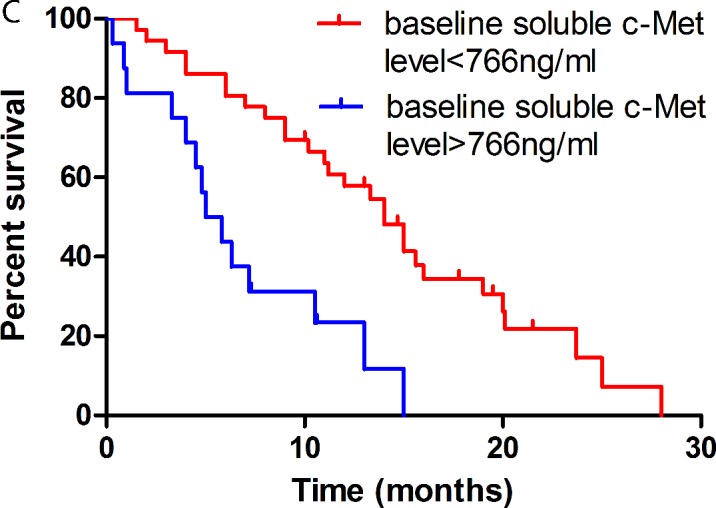

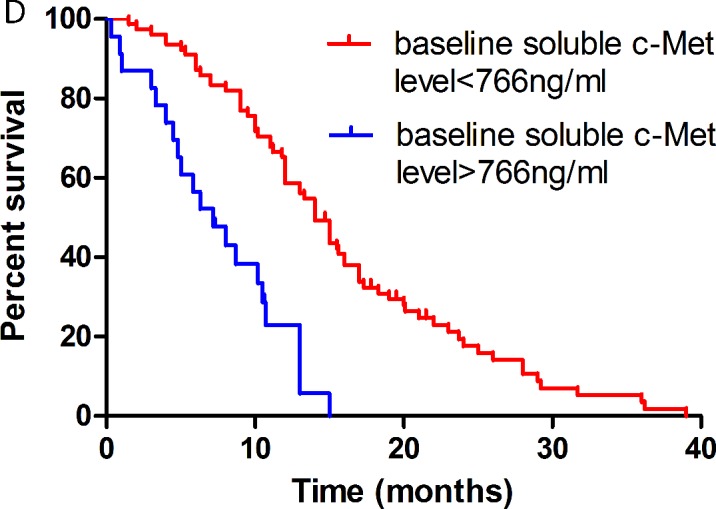
Kaplan-Meier curves of survival analysis after EGFR-TKI treatment according to the level of soluble c-Met (>766 ng/ml *vs* < 766 ng/ml) in pre-EGFR-TKI plasma Notes: **A.** PFS in the training cohort (10.2 *vs* 14.0 months, *P* = 0.003). **B.** OS in the training cohort (20.0 *vs* 33.5 months, *P* = 0.488). **C.** PFS in the validation cohort (6.8 *vs* 14.5 months, *P* < 0.001). **D.** PFS (7.2 *vs* 14.0 months, *P* < 0.001) in the overall patients.

Among the 49 pre-EGFR-TKI plasma samples, the mean plasma c-Met level was 619.83 ± 166.92 ng/ml (range: 318.91 - 1232.67 ng/ml; median: 608.1 ng/ml). Among the 49 patients, 7 (14.3%) had baseline soluble c-Met levels > 766 ng/ml. Patients with soluble c-Met levels > 766 ng/ml showed inferior median PFS after EGFR-TKI treatment (10.2 *vs*. 14.0 months, *P* = 0.003). Patients with soluble c-Met levels > 766 ng/ml were also determined to have inferior overall survival (OS) after EGFR-TKI treatment (20.0 *vs*. 33.5 months,*P* = 0.488); however, this difference was not significant. Multivariate Cox proportional hazards model analyses demonstrated that the soluble c-Met level was an independent prognostic factor for PFS after EGFR-TKI treatment (*P* = 0.009; hazard ratio [HR]: 3.583; 95% CI: 1.379 - 9.312). The above results suggest that soluble c-Met levels pre-EGFR-TKI treatment have potential clinical value as a predictive biomarker for PFS after EGFR-TKI treatment (Figure 3 and Table 3).

The soluble c-Met level in pre-EGFR-TKI plasma test was further assessed in an independent prospectively collected validation cohort of 52 patients (Figure 3). Among the 52 patients, 16 (30.8%) had baseline soluble c-Met levels > 766 ng/ml, 36 (69.2%) with baseline soluble c-Met levels < 766 ng/ml. Patients with soluble c-Met levels > 766 ng/ml were also determined to have significant short median PFS after EGFR-TKI treatment (6.8 *vs*. 14.5 months, *P* < 0.001).

However, there was no significant correlation between plasma c-Met levels at the time of the best response or PD and survival after EGFR-TKI therapy (all P values > 0.05; detailed data not shown).

**Table 3:** Multivariate analyses of prognostic parameters in the training cohort by Cox regression analysis

Characteristic	Multivariate		
	HR	(95% CI)	*P*
Smoking	0.414	(0.107-1.599)	0.201
Soluble c-Met level pre-EGFR-TKI	3.583	(1.379-9.312)	0.009
Age	0.974	(0.440-2.156)	0.949
Sex	1.245	(0.508-3.051)	0.631
EGFR mutation	0.991	(0.481-2.042)	0.981
Best response of EGFR-TKI	0.799	(0.340-1.875)	0.606
Line of EGFR-TKI treatment	1.639	(0.790-3.402)	0.185
Tissue c-Met status with PD	1.526	(0.721-3.229)	0.270

## DISCUSSION

To the best of our knowledge, the present study was the first to discuss the relationship between EGFR-TKI treatment and variations in the soluble c-Met level during certain time points. c-Met is a promising therapeutic target in NSCLC. The expression of c-Met in primary and resistant tumors is essential for EGFR-TKI treatment in advanced NSCLC patients [5, 12]. However, the role of soluble c-Met in the context of the EGFR-TKI setting remains unknown. Several studies have demonstrated a significant correlation between plasma soluble c-Met levels and tissue c-Met protein expression in lung cancer [19, 20]. Our previous study also confirmed this finding and found that a plasma c-Met level of 766 ng/ml showed good specificity and sensitivity in predicting tissue c-Met protein expression. Patients with a high level of soluble c-Met (> 766 ng/ml) had a worse survival time than patients with a low level of soluble c-Met (< 766 ng/ml) (detailed data not shown). In the present study, we determined the c-Met status in matched plasma and tumor tissues with PD and obtained 79.6% concordance and 89.2% specificity. The results presented herein together with previous results suggest the availability of plasma for c-Met detection to offer a promising strategy for monitoring c-Met status during EGFR-TKI treatment.

In our study, we measured the dynamic change in soluble c-Met in plasma during EGFR-TKI treatment. To identify the effect of soluble c-Met levels on EGFR-TKI resistance, we divided the 49 patients into two groups according to c-Met status in the resistant tumor tissue. The average plasma c-Met levels in the tissue c-Met-negative group were 602.72 ± 135.59 ng/ml (95% CI: 403.42 - 873.86) before EGFR-TKI treatment, 531.47 ± 128.85 ng/ml (95% CI: 333.44 - 755.92) at the time of the best tumor response, and 601.55 ± 120.94 ng/ml (95% CI: 386.24 - 881.84) with PD. The plasma c-Met levels in the tissue c-Met-positive group were 672.57 ± 239.58 ng/ml (95% CI: 318.91 - 1232.67) before EGFR-TKI treatment, 546.15 ± 159.54 ng/ml (95% CI: 360.33 - 971.51) at the time of the best tumor response, and 986.42 ± 578.36 ng/ml (95% CI: 483.36 - 2208.06) with PD. There was a statistically significant difference in the dynamic change in the soluble c-Met level between the tissue c-Met-positive and c-Met-negative groups (*P* = 0.002). During EGFR-TKI treatment, we observed that the plasma c-Met level decreased at the time of the best tumor response and increased with PD, particularly in EGFR-TKI-resistant patients with c-Met positivity. Our results suggest that the c-Met signaling pathway might play a role in the response to EGFR-TKI treatment. Treating NSCLC with EGFR-TKI would enrich c-Met-positive tumor cells, with the overexpression and overactivation of c-Met consequently triggering the activation of Her3, thus activating downstream signal transduction molecules such as Akt and Erk, independent of EGFR kinase activity [4, 5], and finally switching to the c-Met pathway to control tumor growth [21]. Because the activation of c-Met signaling contributes to EGFR-TKI resistance and leads to the rapid evolution of drug resistance, strategies for overcoming acquired resistance to EGFR-TKI are now undergoing clinical evaluation; therefore, stratifying patients based on the dynamic change in soluble c-Met during EGFR-TKI treatment might predict the resistance to EGFR-TKI and allow for individualized treatment.

In advanced NSCLC, the relationship between soluble c-Met levels and prognosis after EGFR-TKI treatment remains unclear. Several studies have detected the coexistence of c-Met dysregulation and EGFR gene mutations in tumor tissues of NSCLC patients, and demonstrated that the PFS of patients with both EGFR mutations and c-Met gene amplification was significantly shorter than in patients with EGFR mutations alone [22, 23]. By utilizing ELISA to analyze the c-Met status in plasma, we demonstrated that soluble c-Met levels pre-EGFR-TKI have potential clinical value as a predictive biomarker for PFS after EGFR-TKI. In our study, the baseline soluble c-Met level functioned as a negative prognostic marker in EGFR-TKI treatment. Kaplan-Meier survival analysis demonstrated that patients with high soluble c-Met levels (> 766 ng/ml) before EGFR-TKI treatment had a shorter PFS (10.2 *vs*. 14.0 months; *P* = 0.003). Multivariate Cox proportional hazards model analyses demonstrated that the soluble c-Met level was an independent prognostic factor for PFS after EGFR-TKI (*P* = 0.009; HR: 3.583; 95% CI: 1.379 - 9.312). However, there was no significant correlation between plasma c-Met levels at the time of the best response or PD and PFS after EGFR-TKI therapy. This might be because c-Met-activated tumor cells could presumably proliferate despite EGFR-TKI therapy, and initial disease control was followed by a relatively short PFS compared with tumors without c-Met activation. Taken together, our findings highlight the role of soluble c-Met quantitative analysis in predicting PFS after EGFR-TKI, and might support early administration of a combination of c-Met and EGFR inhibitor therapies. Based on these considerations, our findings form the basis for further studies that may lead to an improvement in current treatment strategies.

Owing to the small sample size, we further assessed the prognosis of soluble c-Met level in an independent validation cohort of 52 patients (Table 1). In the validation set, 16 (30.8%) had baseline soluble c-Met levels > 766 ng/ml, 36 (69.2%) with baseline soluble c-Met levels < 766 ng/ml. Patients with soluble c-Met levels > 766 ng/ml were also determined to have significant short median PFS after EGFR-TKI treatment (6.8 *vs*. 14.5 months, *P* < 0.001). It provided a highly significant proof of principle.

One potential drawback of our study was that c-Met status was not determined before EGFR-TKI treatment because the tissue samples were not available at this time point. Therefore, we could not determine whether a soluble c-Met level > 766 ng/ml before EGFR-TKI treatment indicated that tissue c-Met protein was expressed. Owing to the small sample size, more studies are warranted to confirm these results in the future.

In conclusion, our results demonstrate that the measurement of soluble c-Met using ELISA is feasible and noninvasive with potential clinical applications. Stratifying EGFR mutation NSCLC patients based on the dynamic change of soluble c-Met during EGFR-TKI treatment might allow for individualized treatment. Moreover, tumors with a high soluble c-Met level before EGFR-TKI therapy could exhibit a shorter PFS after EGFR-TKI. Dynamic monitoring of the change in c-Met level might help determine EGFR-TKI prognosis.

## PATIENTS AND METHODS

Between February 2014 and May 2015, we retrospectively analyzed patients with advanced NSCLC (stage IV, PS 0-1) at the Guangdong Lung Cancer Institute who were resistant to EGFR-TKI treatment. The inclusion criteria were as follows: 1) patients being screened for the c-Met inhibitor trial and 2) sufficient plasma samples available for the analysis of soluble c-Met level before EGFR-TKI treatment, at the time of the best response, and PD. We collected plasma and tissue samples with PD, but subsequent treatment was not initiated. The interval time between PD after EGFR-TKI and plasma extract preparation was less than 21 days. PFS after EGFR-TKI was defined as the time interval between the beginning of EGFR-TKI treatment and PD or death. OS was defined as the time interval between the beginning of EGFR-TKI treatment and death. The clinical data were reviewed. The objective tumor response was evaluated by RECIST 1.1 at 8-week intervals, or as clinically indicated, until PD, death, or loss to follow-up. This study was approved by the local Institutional Review Board. All patients provided written informed consent before the collection and use of their tumor specimens and blood samples.

### IHC of c-Met in tumor tissue

The expression of c-Met protein in formalin-fixed paraffin-embedded tissues collected with PD was determined by IHC analysis using an anti-total c-Met rabbit monoclonal primary antibody (SP44; catalog # 7904430; Ventana Medical Systems, Tucson, AZ, USA). IHC staining was carried out according to the manufacturer's protocol using the BenchMark XT platform from Ventana and the ultraView detection kit. Tissue c-Met expression levels were evaluated by IHC according to H score criteria. The expression of c-Met in tumors was defined as follows: no c-Met expression (scored as IHC = 0), weak c-Met expression (scored as IHC = 1+), moderate c-Met expression (scored as IHC = 2+), and strong c-Met expression (scored as IHC = 3+). The fraction of positive cells at each intensity level was estimated as a percentage (0-100%). The H score was the cross-product of the intensity and fraction score, ranging from 0 to 300. Positivity was defined as having ≥50% of tumor cells positive for membranous and/or c-Met immunostaining with strong intensity (IHC ≥3+).

### Soluble c-Met levels in plasma samples

The plasma soluble c-Met protein concentration was measured using a human soluble c-Met quantitative ELISA kit according to the manufacturer's instructions (Invitrogen Corporation, Camarillo, CA, USA).

### ELISA analysis of independent validation cohort

Blood samples were obtained from a consecutive prospective series of advanced NSCLC patients with EGFR mutation treated with EGFR TKI at the Guangdong Lung Cancer Institute from February 2013 to May 2014. Samples were assessed by ELISA blinded to clinicopathologic characteristics.

### Statistical analysis

All analyses were performed using SPSS 16.0. Soluble c-Met changes over time were analyzed using repeated measures ANOVA. The associations between c-Met and clinical parameters were evaluated using the χ^2^ and/or *t*-test. To evaluate the duration of response, Kaplan-Meier estimation was used, and comparisons were made using the log-rank test. Multivariate analysis of categorical variables was performed using logistic regression. A two-sided P value less than 0.05 was considered to be statistically significant.
